# AI in Membrane Design and Optimization for Hydrogen Fuel Cells

**DOI:** 10.3390/membranes16030097

**Published:** 2026-03-03

**Authors:** Bshaer Nasser, Hisham Kazim, Moin Sabri, Muhammad Tawalbeh, Amani Al-Othman

**Affiliations:** 1 Sustainable Energy & Power Systems Research Centre, Research Institute of Sciences and Engineering (RISE), University of Sharjah, Sharjah P.O. Box 27272, United Arab Emirates; bshaer.ali@sharjah.ac.ae (B.N.); mtawalbeh@sharjah.ac.ae (M.T.); 2 Sustainable and Renewable Energy Engineering Department, University of Sharjah, Sharjah P.O. Box 27272, United Arab Emirates; 3 Department of Computer Science and Engineering, American University of Sharjah, Sharjah P.O. Box 26666, United Arab Emirates; b00084686@aus.edu (H.K.); b00084704@alumni.aus.edu (M.S.); 4 Department of Physics, American University of Sharjah, Sharjah P.O. Box 26666, United Arab Emirates; 5 Quantum Research Center, Technology Innovation Institute, Abu Dhabi P.O. Box 9639, United Arab Emirates; 6 Department of Chemical and Biological Engineering, American University of Sharjah, Sharjah P.O. Box 26666, United Arab Emirates; 7 Energy, Water and Sustainable Environment Research Center, American University of Sharjah, Sharjah P.O. Box 26666, United Arab Emirates

**Keywords:** hydrogen energy, PEM fuel cells, membrane design, performance optimization, AI prediction model

## Abstract

This paper reviews artificial intelligence (AI) applications in the design and optimization of proton exchange membrane (PEM) materials for hydrogen fuel cells. Clean energy conversion is a substantial benefit of PEM fuel cells, which conventional membrane development struggles with due to time-consuming trial-and-error methods, which are not adequate in capturing the different interdependencies of the membrane structure, and environmental variables. The review establishes foundational design principles of PEMs and outlines their challenges and computational methodologies are constructed to address them. Various advanced AI methods have been highlighted which include graph neural networks, multitask frameworks, and physics-informed models that facilitate rapid prediction of polymer properties. Optimization methods have been reported with 10–30% performance improvements, for instance, NSGA-II frameworks achieving 13–27% gains in power density. Experimental requirements are reduced by 40–60%, as seen with Bayesian optimization, identifying optimal designs within as few as 40 iterations. Current challenges include data availability, generalizability, and scalability, which are closely assessed in this review.

## 1. Introduction

The global transition toward low-carbon and sustainable energy systems has accelerated global interest in hydrogen as a clean energy carrier among the other available options, given its ability to significantly support decarbonization across multiple sectors [1]. Hydrogen can be produced through a variety of production methods and pathways, and the cleanest one is water electrolysis powered by renewable electricity [2]. It offers a unique combination of high energy density and zero- to low-carbon energy conversion and, hence, strong potential for carbon-neutral production and zero carbon emissions at the point of use. This will position it as a key pillar of future clean energy infrastructures. Once produced, hydrogen can be efficiently converted into usable energy [3]. Notably, fuel cells represent effective and clean technologies for this purpose, as they directly transform the chemical energy stored in hydrogen into electrical energy through electrochemical reactions. Numerous types of fuel cells have been developed, distinguished primarily by the nature of their electrolyte and operating temperature conditions. These include alkaline fuel cells, phosphoric acid fuel cells, molten carbonate fuel cells, solid oxide fuel cells, and proton exchange membrane fuel cells (PEMFCs) [4,5]. PEMFCs have emerged as one of the most mature and technologically advanced types. This is mainly due to their high efficiency, high power density, relatively low operating temperatures typically below 90 °C, fast start-up, quiet operation, all-solid structure, and compatibility with intermittent operation [2,6]. These characteristics make them well suited and ideal for transportation, stationary power generation, and portable applications [7]. A PEMFC is composed of several components that work together to achieve efficient electrochemical conversion. These components are mainly the bipolar plates (BPs), gas diffusion layers (GDLs), catalyst layers (CLs), and the electrolyte, which is simply known as the proton exchange membrane or the polymer electrolyte membrane (PEM). While each component contributes to the overall system performance, the PEM occupies a central and uniquely critical role in the electrochemical reaction in terms of conducting protons, blocking electrons from crossing from the anode to the cathode, and preventing reactant gases from mixing (crossover) [8]. Historically, perfluorosulfonic acid (PFSA) membranes, most notably Nafion material, have dominated the PEMFC technology field because of their high proton conductivity and chemical stability. However, these membranes face inherent limitations such as their high material cost, significant performance degradation at elevated temperatures or low humidity, and environmental concerns regarding these fluorinated polymers [9]. In response, extensive research efforts have focused on polymer membrane chemistry aimed at improving membrane conductivity, stability, and durability while reducing cost and environmental impact. These advances reflect and highlight the importance of membrane design as a multidimensional optimization process rather than a single property enhancement task. Membrane structure, composition, and interaction with surrounding components are considered as an integrated system rather than isolated features [10,11]. Traditional membrane development approaches have relied heavily on experimental trial-and-error methods. While these strategies have yielded important advances, they are inherently time-consuming, resource-intensive, and limited in their ability to capture the complex interdependencies between membrane structure, operating conditions, and performance outcomes [12]. In this context, recent advances in predictive modeling, optimization, and data-driven techniques, including artificial intelligence (AI) and machine learning (ML), are increasingly being explored as complementary tools for membrane research. These approaches offer the potential to identify hidden correlations, predict membrane behavior under diverse operating conditions, and guide rational design strategies more efficiently than conventional methods alone [13,14]. Despite that, the successful application of such techniques depends fundamentally on a clear and accurate understanding of membrane design principles, functional requirements, and performance-limiting mechanisms [15].

This work establishes and identifies the fundamental design principles, properties, and operational challenges of PEMs. It also focuses primarily on the scientific basis upon which advanced predictive and optimization AI approaches in PEMs research can be built. By comprehensively examining membrane behavior, degradation mechanisms, and design constraints, this study provides a coherent framework that supports the integration of emerging computational and data-driven methodologies in reshaping how membrane materials are evaluated and optimized to identify optimal membrane designs. To the best of the authors’ knowledge, this has not yet been reported in the literature.

## 2. Background on Hydrogen Fuel Cells

**Figure 1:**
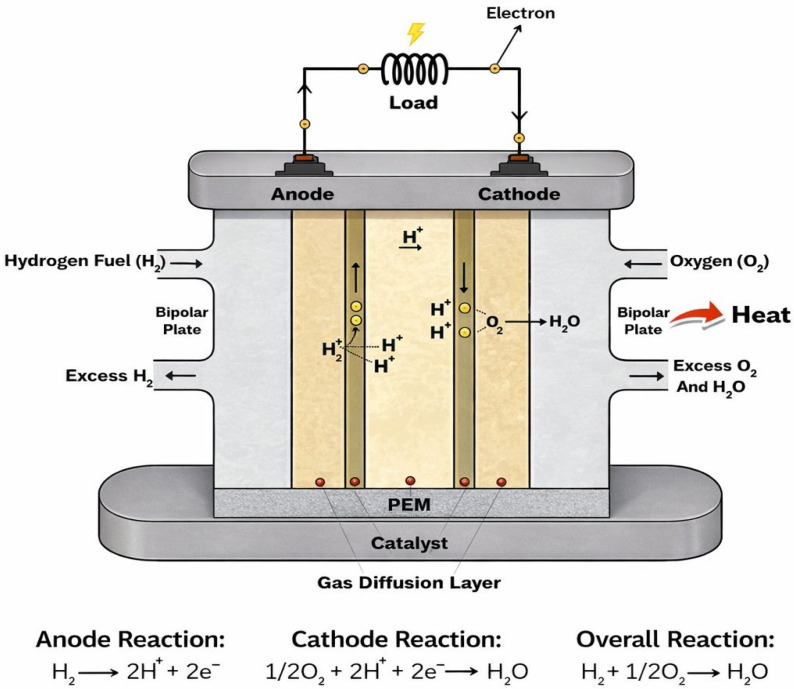
PEM fuel cell operation.

Unlike combustion-based energy systems, fuel cells rely on electrochemical reactions that have higher theoretical efficiencies and cleaner operation. Hydrogen fuel cells operate through the direct electrochemical conversion of hydrogen and oxygen into electrical energy, heat, and water as the only reaction products [2]. At the core of this device lies the PEM itself, which serves as a solid electrolyte and plays a multifunctional role that extends far beyond simple ionic conduction. Within the fuel cell structure, the membrane is integrated into the membrane electrode assembly (MEA), where it is sandwiched between two CLs [16]. Acting as the electrolyte, the PEM separates the anode and cathode plates while allowing selective transport of protons from the anode to the cathode. At the same time, it blocks electrons from passing through it and prevents direct mixing of reactant gases such as hydrogen and oxygen. As such, PEM serves not only as an ionic conductor but also as a physical, chemical, and mechanical barrier within the fuel cell [8]. Basically, during the operation, hydrogen is supplied to the anode, where it undergoes electrochemical oxidation, releasing protons and electrons. The electrons are forced through an external circuit to generate direct electrical current, while the protons migrate internally through the PEM to the cathode. At the cathode, oxygen reduction occurs to combine oxygen molecules with protons and electrons to form water, as seen in Figure 1 [17].

Although CLs facilitate these electrochemical reactions, the membrane determines how effectively protons are transported between them and how well reactants remain separated. This dual function of selective ion transport and gas barrier performance is fundamental to fuel cell efficiency and operational safety. Beyond its electrochemical function, the membrane exerts strong control over the internal physicochemical environment of the fuel cell. Its interaction with water, gases, and adjacent porous layers directly influences local reaction conditions and transport pathways. During operation, the membrane is exposed to changes in temperature, humidity, and electrochemical potential, which are harsh and vary across the cell with load demand [18]. These gradients induce coupled transport processes involving charge species, such as protons and electrons, water, and heat, all of which must be accommodated by the membrane without loss of functionality. Membranes may function under harsh operating environments; these operating conditions may further complicate membrane behavior. Over time, repeated exposure to such conditions can lead to irreversible changes in membrane structure [19]. Accordingly, the following sections focus on the key properties that define membrane functionality, the material strategies employed to achieve these properties, as well as the inherent trade-offs that arise during membrane design.

## 3. Membrane Operational Performance Properties and Material Design

### 3.1. Key Membrane Properties and Parameters

**Figure 2:**
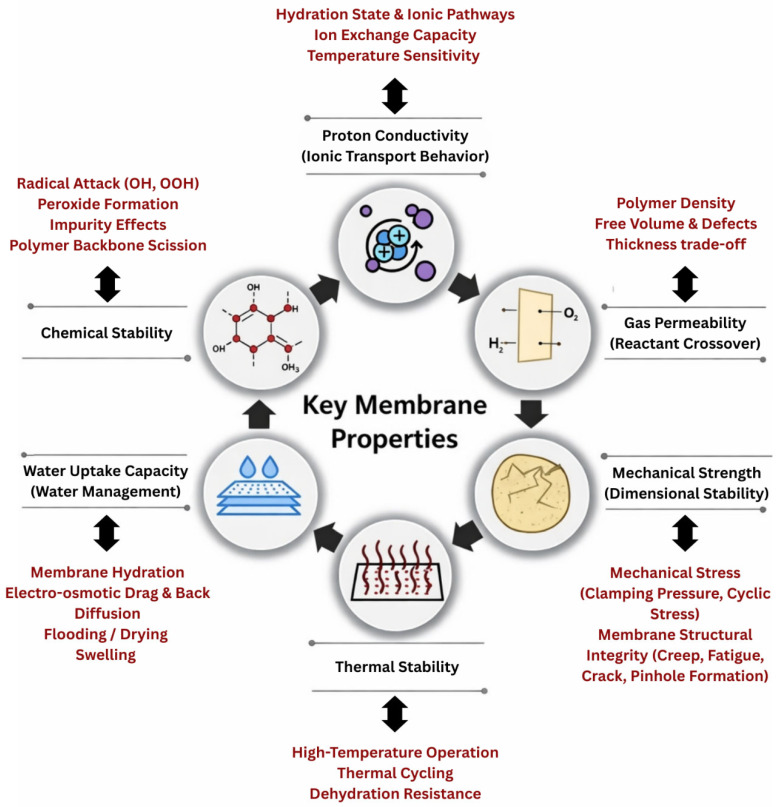
Key membrane properties and their associated mechanisms in PEMFC operation.

The performance and durability of PEMFCs are primarily dependent on the intrinsic and operational properties of the PEM [20]. Far from being a passive separator, the PEM functions as a dynamic material whose behavior evolves under the influence of coupled electrochemical, thermal, and mechanical factors [10,21]. Complex transport phenomena exist at the membrane. Therefore, membrane functionality is strongly modulated by its interaction with adjacent components such as the CLs, GDLs, and BPs [8,22]. These interfaces directly shape the ion transport pathways, local hydration states, and mechanical constraints. Although the membrane is supported by these surrounding layers, it is also constrained by them. Basically, CLs affect interfacial contact and ion transport pathways through the porous media, while GDLs and microporous layers influence the distribution of reactants that impact membrane functionality [23]. This means membrane properties must be understood as interconnected parameters within a complex system that determines fuel cell efficiency, operational reliability, stability, and lifetime. During operation, the PEM experiences electrochemical potential gradients, thermal cycling, mechanical stresses, and chemically harsh environments due to the formation of radicals. Similarly, modern PEMFCs are increasingly expected to perform and operate under dynamic loads, lower external humidification, and harsher thermal environments, placing additional demands on membrane robustness and adaptability [24]. Hence, adopting a membrane-centric perspective provides a framework for a comprehensive understanding of how material-level design choices translate into device-level performance [11]. In practice, the PEM must satisfy several critical design properties and requirements while operating often under normal, harsh, and complex conditions, as illustrated in Figure 2. The prioritization of proton conductivity, gas permeability, thermal and chemical stability, mechanical strength, and water management is based on both fuel cell electrochemical theory and extensive experimental durability and degradation studies [25,26,27,28]. Proton conductivity directly controls ionic transport, ohmic losses and power density, while gas crossover controls fuel utilization efficiency and chemical degradation rates. Thermal and chemical stability determine membrane lifetime under elevated temperature and radical-rich environments, whereas mechanical integrity determines resistance to cyclic hydration stress, fatigue, creep, and pinhole formation. Finally, water uptake and internal water management regulate the balance between ionic transport, dimensional stability, and electrode flooding or drying [29,30,31]. Together, these properties directly define the efficiency, durability, and safety of PEMs and are therefore universally recognized as the primary design constraints in PEMFC performance and lifetime. Designing a membrane that can simultaneously satisfy all of these requirements remains one of the most significant scientific and engineering challenges in PEMFC development. The primary challenge lies not in identifying these individual relationships but in quantitatively predicting their synergistic effects across a broad design area.

#### 3.1.1. Proton Conductivity and Ionic Transport Behavior

Proton conductivity is the defining functional property of PEMs in fuel cells, as it directly controls the ionic transport between the anode and cathode and thus determines cell efficiency and power density [32]. At the molecular level, proton transport occurs within hydrated ionic domains facilitated by water molecules that form hydrogen-bonded networks. Proton transport is known to occur by two mechanisms, either vehicular diffusion, where protons migrate with water molecules, or the Grotthuss mechanism, which involves protons hopping from one water molecule to another through the hydrogen-bonding network. The efficiency of this process depends on both the availability and density of proton-conducting sites within the membrane’s internal structure [32]. Achieving high proton conductivity requires precise control of water content, which is crucial, along with polymer chemistry, ionic group density, and microstructural bonding to ensure continuous proton-conducting pathways. Therefore, conductivity is highly sensitive to hydration state and temperature operating conditions. This sensitivity poses a major challenge for PEM fuel cell operation, particularly under low-humidity or high-temperature environments, where maintaining sufficient conductivity without external humidification becomes difficult. Increasing ionic group concentration or water uptake can improve proton transport, but such strategies often induce excessive swelling, reduced mechanical strength, and accelerated chemical degradation [33], whereas operating under dry conditions sharply decreases the conductivity of the membrane [8]. In short, the enhancement of proton conductivity often involves trade-offs with other membrane properties. As a result, proton conductivity cannot be maximized in isolation and must be optimized in conjunction with morphology, structural, and stability considerations of the membrane. This highlights its central nature yet interdependent role in membrane design.

#### 3.1.2. Gas Permeability and Reactant Crossover

In addition to enabling proton transport, the membrane must effectively prevent the direct mixing of hydrogen and oxygen through it. Gas permeability, specifically hydrogen and oxygen crossover, is a critical parameter that influences both the efficiency and the safety of the fuel cells [34]. This property is mainly influenced by membrane thickness, polymer density, and microstructural features such as free volume and defects. Excessive hydrogen crossover, where fuel permeates through the membrane, not only lowers fuel utilization efficiency by reducing open-circuit voltage (OCV) and increasing losses but also accelerates membrane chemical degradation through the formation of reactive oxygen species (ROS) within the membrane [8,35]. A primary design conflict that exists regarding membrane thickness is that thinner membranes generally exhibit lower ohmic resistance and higher power density, but they are more exposed to gas crossover and mechanical failure. Nevertheless, thicker membranes offer superior barrier properties at the expense of increased ohmic losses. Thus, thinner membranes amplify this problem, forcing designers to choose between low ionic resistance and acceptable gas barrier performance. In response, gas crossover presents an additional limitation that becomes increasingly significant as membrane thickness is reduced [36]. New composite membranes and modified polymer structures are often explored to prevent gas diffusion while preserving proton transport pathways. These approaches may introduce additional complexity in membrane design and highlight the need for predictive understanding of how microstructural features affect transport phenomena [37]. This trade-off represents the difficulty of independently optimizing membrane thickness, transport behavior, and durability using conventional design approaches, where improvements in one performance metric often come at the cost of another.

#### 3.1.3. Thermal and Chemical Stability

During fuel cell operation, membranes are exposed to elevated temperatures and fluctuating humidity levels. It was found that thermal stability becomes increasingly important as fuel cells are pushed toward higher operating temperatures to improve reaction kinetics and water management. While higher temperatures can enhance proton transport and reduce catalyst poisoning, they also accelerate chemical degradation processes within the membrane. Therefore, membrane materials must be designed to withstand prolonged exposure to elevated temperatures without significant loss of structural or functional integrity, such as melting, dehydration, shrinkage, or rupture [38,39]. Over extended operating periods, these conditions can induce chemical degradation of polymer backbones, side-chain cleavage, and loss of functional groups, ultimately leading to decreased conductivity and mechanical failure [36]. Hence, chemical stability is closely linked to membrane composition and the nature of its functional groups. In these aggressive chemical environments, to resist this type of degradation, its causes must be investigated and considered. This degradation is primarily due to the formation of highly reactive radical species, such as hydroxyl (OH) and hydroperoxyl (OOH) radicals, which originate from hydrogen peroxide (H_2_O_2_) intermediates generated during oxygen reduction cathode and fuel crossover reactions across the membrane, especially under high potential or transient operating conditions. Once formed, these radicals can attack polymer backbones and side chains, resulting in chain scission, loss of functional groups, pinhole formation, defects, and progressive decline in proton conductivity [30,40]. In addition, chemical degradation is significantly accelerated by the presence of trace impurities and metal contaminants, which may originate from catalysts, system components, or reactant gases. Such impurities can substantially increase fluoride emission rates, which can further destabilize the membrane’s chemical and mechanical structure [40,41]. Although fluorinated membranes exhibit relatively superior resistance to oxidative attacks, which explains their widespread adoption, they are not protected or preserved against long-term degradation. Moreover, some concerns related to their cost and environmental impact have motivated the exploration of alternative materials, many of which exhibit lower chemical robustness. Non-fluorinated and modified membranes, while attractive from a cost and sustainability perspective, often exhibit an even greater chance of chemical attacks. This creates a design dilemma in which chemically robust materials may be costly or environmentally problematic, while more sustainable alternatives struggle to meet durability requirements [8,42]. In addition, integrating radical scavengers, reinforced backbones, or inorganic fillers to modify these membranes can mitigate these effects but may introduce new complexities in transport morphology. In order to guide rational membrane design, it is important to identify the parameters behind these degradation mechanisms.

#### 3.1.4. Mechanical Strength and Dimensional Stability

The mechanical strength and dimensional stability of a membrane refer to its ability to withstand stress without damage. During operation, the membrane experiences continuous mechanical loading due to cyclic hydration, temperature changes, and compressive stress (clamping pressure) within the fuel cell stack. Over time, these stresses can exceed the membrane’s mechanical tolerance [30]. Structurally, this results in repeated volumetric expansion and contraction, which generates internal stresses leading to creep, fatigue, and pinhole formation. Mechanical strength of a membrane is influenced by polymer backbone rigidity, degree of crystallinity, reinforcement strategies, as well as membrane thickness. These are critical factors for maintaining membrane integrity during fuel cell operation. There is a fundamental tension between flexibility and rigidity. Highly flexible membranes may better tolerate deformation but are more prone to creep, mechanical failure, fatigue failure, and thinning under compression. In contrast, rigid membranes offer improved dimensional stability but may fracture under cyclic stress. Achieving an optimal balance between flexibility and strength is essential for ensuring long-term durability. Similarly, thinner membranes, which are desirable for reducing ohmic losses, are particularly susceptible to mechanical failure, whereas thicker membranes negatively affect performance due to increased resistance [43]. Furthermore, repeated volumetric expansion and contraction induced by hydration–dehydration cycles generate mechanical stresses that accelerate dimensional changes and instability in the form of crack formation, membrane thinning, delamination from adjacent layers, and pinhole defects. Moreover, the mechanical behavior of the membrane also affects its interaction with other fuel cell components. Poor mechanical compatibility can increase interfacial resistance and reduce effective contact with catalyst layers, indirectly impacting electrochemical performance. Thus, mechanical properties must be considered not only from a structural perspective but also in terms of their impact on transport and interfacial phenomena. In fact, mechanical degradation exacerbates other failure mechanisms like chemical degradation by facilitating gas crossover and creating localized hot spots, demonstrating that mechanical integrity is tightly coupled to transport and chemical stability [36]. The ability of a membrane to accommodate such stresses without losing functionality is therefore a key design requirement. Designing membranes that maintain mechanical resilience without compromising proton conductivity under dynamic operating conditions remains a major unresolved issue in membrane development.

#### 3.1.5. Water Uptake and Internal Water Management Behavior

Water plays a dual role in PEMs; it acts as a facilitator of proton transport [44]. As discussed previously, proton conduction in PEMs is strongly dependent on the hydration level. The membrane should have sufficient water content to maintain proton transport since water molecules facilitate proton transport through both vehicular and Grotthuss mechanisms. Further, water transport involves multiple competing mechanisms such as electro-osmotic drag and back diffusion. Electro-osmotic drag plays a central role in the transport of water molecules as, when the protons migrate from the anode to the cathode, they carry and drag with them the water molecules along the ionic pathways. This transport mechanism leads to water accumulation at the cathode side while gradually dehydrating the anode side, thus drying the membrane and lowering the conductivity as well. On the opposite side, as the concentration of the produced water at the cathode from the electrochemical reaction increases, the water starts to diffuse back toward the anode due to the concentration difference t. This mechanism is known as back diffusion and is driven by the water concentration gradient across the membrane [16,45]. Nevertheless, excessive water uptake must be avoided because it can lead to excessive swelling or water accumulation that disrupts fuel cell operation [45]. Simply, insufficient hydration leads to membrane drying, while excessive water accumulation causes swelling and flooding in adjacent electrode layers. Flooding blocks gas transport pathways and increases interfacial resistance; on the other hand, membrane drying causes sharp reductions in conductivity and increased mechanical brittleness. Therefore, dimensional instability as well as mechanical stress within the membrane can occur as a reaction to this behavior. It was found that water uptake can be determined by chemical microstructure of the membrane, polymer hydrophilicity, ionic group distribution, membrane morphology, etc. [46]. The water content depends on a delicate balance between these competing effects. For instance, membranes with high hydrophilicity exhibit improved hydration but are more susceptible to dimensional instability, while membranes with limited hydrophilicity may suffer from dehydration and conductivity [38]. The membrane must therefore be designed to maintain an optimal and adequate hydration state across a wide range of current densities, temperatures, and humidity conditions. This requirement imposes stringent constraints on membrane chemistry, morphology, thickness, and interfacial compatibility with surrounding layers. Uncontrolled water accumulation or depletion leads to performance losses and accelerated degradation, which has attracted attention to water management strategies. Water management within the membrane further complicates membrane design. Internal water management is therefore a central and complex challenge that links membrane transport behavior, mechanical stability, and overall system performance in PEMFC operation. It is important to note that water management within the membrane cannot be separated from the behavior of the surrounding layers. Water generated at the cathode, transported through the membrane, or removed via gas diffusion layers directly affects membrane hydration [47]. Modern design aims for “self-regulating” water behavior to eliminate the need for heavy external humidification systems. Traditional membrane designs often rely on external humidification or conservative operating windows, both of which add system complexity and reduce overall efficiency.

### 3.2. Main Types of Materials for Membrane Design

From a materials perspective, PEMs are typically composed of a polymer backbone functionalized with acidic groups that facilitate proton dissociation and transport. The internal structure of these membranes, specifically the phase-separated hydrophilic and hydrophobic domains, dictated the ionic pathways and their interactions with water [10]. While the chemical composition of the membrane defines its intrinsic properties, its thickness, morphology, and interfacial compatibility with other fuel cell components determine how these properties manifest under operating conditions [47]. Moreover, this is because membrane performance is not simply a material matter but rather a design problem that extends from molecular-level chemistry to device structure. The classification of PEMs reflects fundamentally different design and modification approaches aimed at balancing ionic transport, stability, durability, and cost [7]. Each membrane class represents a specific approach to addressing the coupled properties and degradation challenges discussed in the previous section. From a membrane design perspective, understanding these classes is essential because each type embodies distinct structure–property relationships that directly influence fuel cell operation and lifetime. Rather than focusing on fabrication routes, this section examines membrane types through the lens of functional design and operational performance.

#### 3.2.1. Perfluorosulfonic Acid Membranes

PFSA membranes represent the most established and extensively studied class of polymer electrolytes for PEMFCs. Their widespread adoption is largely attributable to their high proton conductivity, chemical robustness, and well-defined phase-separated morphology. The polymer backbone consists of a hydrophobic fluorinated matrix, while sulfonic acid side chains form hydrophilic domains that enable proton transport. This nanoscale phase separation creates continuous ionic pathways under hydrated operating conditions [48]. In fact, their high conductivity is strongly dependent on membrane hydration, so their performance is sensitive to operating temperature and humidity. Their conductivity decreases significantly under low-humidity or elevated-temperature conditions (because of water evaporation). However, excessive humidity will lead to membrane swelling, which in turn causes dimensional instability and mechanical degradation during cyclic operation. The solution revolves around finding the optimum level of humidity [4]. In addition, PFSA membranes are associated with high material cost and environmental concerns related to fluorinated polymers. A representative example of this membrane class is Nafion, which has served not only as a commercial standard material but also as a design baseline in PEMFC development research. Numerous studies have shown that, although PFSA membranes provide excellent initial performance, their long-term durability remains a limiting factor, particularly under harsh conditions. Madhav et al. [30] provided a comprehensive review of PEM degradation pathways, highlighting how hydroxyl and hydroperoxyl radicals formed during operation attack both side and main polymer chains of PFSA, leading to loss of conductivity, increased gas permeability, and mechanical integrity. Additionally, Pratama et al. [49] evaluated the durability of PFSA membranes under combined electrochemical stress and wet–dry cycling, revealing that reactive species generated during fuel cell operation increase gas permeability, accelerate membrane degradation, and reduce cycle performance, particularly in non-reinforced Nafion membranes. Although Nafion-based membranes exhibit excellent performance under well-humidified conditions, their limitations highlight the broader challenge of designing membranes that maintain functionality across a wide range of operating environments [50]. These limitations have driven extensive efforts to modify PFSA membranes or develop alternative materials that retain their desirable properties while mitigating their drawbacks.

#### 3.2.2. Modified Membranes

Modified membranes aim to improve the intrinsic limitations of conventional PFSA materials along with other PEM materials. Design strategies in this category focus on tuning membrane internal structure, chemical composition, transport behavior, and stability through chemical or physical modification [6]. These modifications include blending with other polymers, altering side-chain length, degree of sulfonation, introducing fillers, additives, or incorporating stabilizing functional groups to enhance specific membrane properties like dimensional stability and water retention behavior [19]. Thus, rather than replacing the entire membrane material, modified membranes seek to adjust the structure and property relationships within a defined design range. As such, these membranes serve as transitional solutions that inform the development of more advanced membrane architectures. Studies have shown that controlled modification of polymer chemistry can reduce excessive swelling, enhance resistance to radical attack, and extend membrane lifetime under aggressive operating conditions. Selim et al. [51] developed WO_3_-doped Nafion membranes by incorporating tungsten oxide (WO_3_) nanofillers into the Nafion matrix. The hybrid membrane showed higher water uptake, lower swelling ratio, improved mechanical stability, and better single-cell performance at 80–95 °C compared to commercial Nafion. By restricting polymer chain mobility or reinforcing the membrane matrix, modified PFSA membranes can exhibit improved mechanical strength and reduced degradation under cyclic operating conditions. Li et al. [52] established PFSA membranes reinforced with a novel expanded polytetrafluoroethylene (ePTFE) and natural radical scavengers to evaluate how adding radical scavengers improves PFSA membrane resistance to degradation, showing a decrease in fluoride emission rates and an improvement in long-term stability with high proton conductivity [53]. The key challenge associated with modified membranes lies in the non-linear response of membrane properties to chemical changes. Sometimes, alterations that enhance stability often reduce ionic mobility or increase transport resistance. Small variations in polymer structure can also produce disproportionate effects on conductivity, hydration dynamics, and mechanical behavior.

#### 3.2.3. Composite Membranes

Composite membranes represent a design strategy that integrates multiple material phases to achieve synergistic performance enhancements. These membranes typically combine a polymer matrix with inorganic or nanostructured fillers, such as oxides, carbon-based materials, or functional nanoparticles [54,55]. The objective is not simply to add fillers but to engineer interactions between phases that improve specific membrane functions. They are designed to address multiple challenges simultaneously. Vinothkannan et al. [56] fabricated Nafion-based composite PEMs with Fe_3_O_4_–SGO fillers by incorporating iron oxide (Fe_3_O_4_) nanoparticles anchored on sulfonated graphene oxide (SGO) to enhance performance under high-temperature and low-humidity conditions. Their results showed that the composite membranes exhibited improved water retention and higher proton conductivity at 120 °C and 20% relative humidity compared to pristine Nafion. In addition, hydrogen permeability was reduced, indicating enhanced gas barrier properties alongside improved transport performance. In fact, inorganic fillers can enhance mechanical strength, reduce gas permeability, and improve thermal stability, while also influencing water retention and transport pathways. However, the effectiveness of composite membranes depends critically on filler dispersion, interfacial compatibility, and loading level [54]. Poorly designed composites can introduce defects, disrupt ionic pathways, or increase resistance. Park et al. [57] investigated recast Nafion composite membranes containing zirconium dioxide (ZrO_2_)–silicon dioxide (SiO_2_) binary oxide fillers with varying Zr/Si ratios for high-temperature PEMFC (HT-PEMFC) operation above 100 °C. Experimental results showed that all composite membranes exhibited higher water uptake than pristine Nafion, while proton conductivity increased with zirconia content at 80 °C but decreased at 120 °C. Hence, these membranes exemplify the shift from single-material optimization toward multiphase design strategies. They also generate complex datasets relating composition, microstructure, and performance, which later can naturally connect to predictive and optimization approaches.

### 3.3. Optimization Strategies for Membrane Material Design

As demonstrated in the previous section, these three different materials represent fundamentally different membrane design areas with increasing levels of structural, physical, and chemical complexity. Table 1 summarizes the main differences between these three types of membranes in terms of design material properties. Understanding their design and properties is used to motivate and guide the optimization processes which offer a systematic route for accelerating membrane material discovery and rational design. Since each membrane class has different design variables, their optimization targets are different, which influences how optimization strategies are applied [58,59,60].

From an optimization perspective, PFSA membranes rely on the chemistry of fluorinated polymers with relatively constrained molecular structures and well-defined structure–property relationships. Consequently, their optimization largely depends on accurately adjusting the equivalent weight, membrane thickness, side-chain length, and hydration behavior within a comparatively narrow range of parameters. In contrast, modified membranes add additional chemical and morphological variables, such as degree of sulfonation, additive incorporation, polymer blending ratio, crosslinking density, etc. These modifications generate complex non-linear relationships between the membrane’s chemistry, morphology, transport behavior, and durability, where even small structural changes can lead to significant performance variations [61]. As a result, the range of potential improvements and optimization becomes much broader and more difficult to capture with simple empirical parametric studies. Composite membranes represent the highest levels of design complexity by incorporating multiphase materials, where membrane performance emerges from coupled interactions between polymer matrices, inorganic or nanofillers, and interfacial transport phenomena. In these systems, membrane properties are influenced not only by the composition but also by filler dispersion, interfacial compatibility, loading fraction and multiphase transport pathways. This results in a high-dimensional and multiparameter optimization design area with highly interconnected and competing objectives [62].

Although PFSA, modified, and composite membranes differ in composition and structural complexity, they share a common design challenge: membrane performance arises from interdependent physical and chemical processes that cannot be optimized independently. Thus, these improvements often introduce new trade-offs between conductivity, durability, gas permeability, and stability, which must be carefully balanced through rational design.

**Table 1:** Comparison of PEM types.

Membrane Type	Core Membrane Design Concept	Key Improvement	Principal Limitations	Ref.
PFSA Membranes	Designed to provide high proton conductivity and chemical/mechanical stability in acidic and hydrated environments. Acts as the industry benchmark for PEMFCs (e.g., Nafion).	- High proton conductivity - Strong chemical stability - Long operational lifetime	- High cost - Humidity dependence (limited conductivity under dry or high-temperature conditions) - Environmental concerns	[9,63]
Modified Membranes	Developed to extend PFSA usability at higher temperatures and lower humidity by introducing fillers or chemical modifications (e.g., sulfonated, crosslinked, or doped variants).	- Enhanced mechanical stability - Improved thermal stability and proton conduction under dry conditions - Good water retention - Reduced gas crossover	- Added complexity - Potential conductivity loss and phase separation - High cost - Less flexible	[19,64]
Composite Membranes	Created to combine PFSA or hydrocarbon polymers with inorganic or nanoporous materials like SiO_2_, ZrO_2_, GO, MOFs, etc. to merge strength, stability, and conductivity.	- Enhanced mechanical strength and chemical resistance - Lowed fuel permeability - Tunable properties	- Interfacial resistance - Complexity - Dispersion challenges - Filler loading	[54,55,65]

### 3.4. Current Status in Membrane Design Technologies

**Figure 3:**
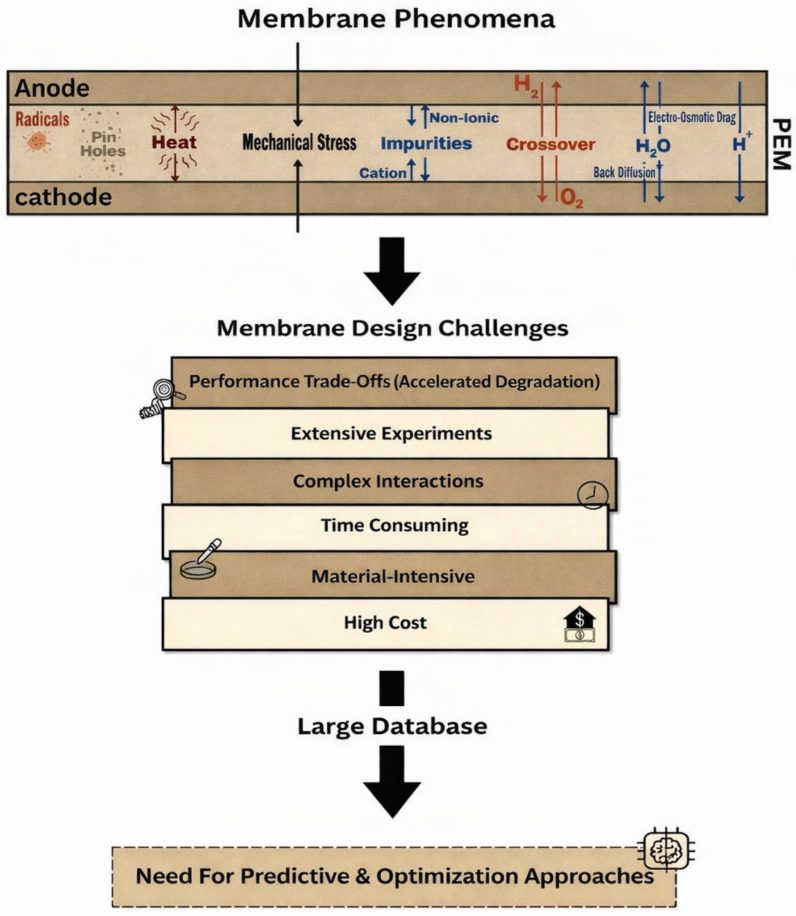
Membrane design challenges.

Despite decades of intensive research and advances in membrane materials development and fuel cell engineering, the design of PEMs that simultaneously satisfy performance, durability, and cost requirements remains a central challenge in hydrogen fuel cell technology [11]. As shown in Figure 3, this challenge associated with membrane technologies does not arise from a lack of fundamental understanding of individual membrane properties or a lack of suitable materials alone but rather from the complex and often competing requirements imposed on membranes during fuel cell operations [10,66]. These challenges are multidimensional, involving coupled transport phenomena, mechanical, chemical degradation processes, economic constraints, etc. Thus, membrane limitations must be understood as design trade-offs rather than isolated material deficiencies and issues.

One of the most fundamental challenges in membrane design technology is the successful coupling between proton conductivity and membrane stability. This lies in the strong interdependence between key membrane properties, where improving one property often leads to degradation of another [67]. For example, high proton conductivity is typically achieved by increasing the density or the concentration of ionic functional groups and enhancing membrane hydration, both of which facilitate proton mobility via vehicular and Grotthuss mechanisms. These same design choices often simultaneously cause excessive water uptake, which can result in excessive swelling, mechanical stress, and accelerated chemical degradation [36]. This fundamental trade-off restricts the extent to which conductivity can be enhanced without compromising membrane durability.

Traditional membrane design relies heavily on experimental iteration, where material composition, functionalization degree, membrane thickness, and processing conditions are systematically varied and evaluated through electrochemical and durability testing. Although this approach has achieved gradual improvements, it is by its nature time-consuming and resource-intensive and can be very expensive [68] since each design iteration requires membrane synthesis, casting, and multiparameter testing under controlled fuel cell operating conditions. When multiple properties must be optimized simultaneously, the number of required experiments increases exponentially. This makes the comprehensive exploration of the membrane design impractical [13]. As a result, many potentially promising membrane compositions and structures remain unexplored simply due to experimental constraints.

Furthermore, this dependence on traditional experiment-based membrane development methodologies typically involves modifying one parameter at a time and evaluating its effect on a limited set of performance metrics. While such methods provide valuable insights, they are inherently slow and poorly suited to capturing the complex, non-linear interactions between membrane composition, structure, operating conditions, and performance outcomes [69]. Here, the design process is further complicated by the need to take into account the real operating conditions that vary dynamically over time. The PEMs experience continuous fluctuations in temperature, humidity, current density, and load cycles during operation; all of these influence hydration state, mechanical stress, and degradation pathways [70]. Traditional experimental studies often isolate individual parameters under steady-state conditions, providing valuable but incomplete insights. As membrane systems become more complex, the number of relevant design variables increases rapidly. Monitoring the combined effects of dynamic operating conditions on these design variables requires long-term, multifactor tests that are not only expensive but also difficult to reproduce consistently.

Cost represents another critical issue that is closely tied to membrane design strategies. High-performance PEMs often rely on expensive fluorinated polymers or raw materials and complex synthesis routes or specialized additives; all of which contribute significantly to the overall cost of fuel cell systems and hinder large-scale deployment. Attempts to reduce material cost by using alternative polymers or simplified fabrication techniques frequently result in compromised durability or performance [71,72]. Moreover, the cost of membrane development itself, including materials, equipment, and long-term testing, adds an additional layer of economic pressure. These factors combined limit the scalability of conventional membrane optimization approaches and slow the conversion of laboratory-scale innovations into commercially viable technologies. Material availability introduces further constraints that shape membrane development strategies. While alternative materials have been proposed, integrating them into PEMs without sacrificing performance remains a significant challenge [62]. Consequently, membrane design must increasingly account for cost and scalability alongside electrochemical performance.

These non-linear trade-offs make it extremely insufficient and difficult to optimize membrane performance using conventional trial-and-error approaches, as the effect of a single design variable cannot be evaluated independently of the others. Consequently, optimization through experimental trial-and-error becomes increasingly inefficient as the number of adjustable parameters grows. As experimental datasets continue to grow in size, complexity, and failure modes, there is an increasing need for systematic frameworks capable of integrating diverse membrane properties, predicting hidden correlations, and guiding targeted optimization. Addressing these challenges requires moving beyond trial-and-error methodologies toward predictive and data-driven approaches that can accelerate membrane design innovation while reducing experimental load [73].

## 4. The Need for AI in Membrane Material Design

**Figure 4:**
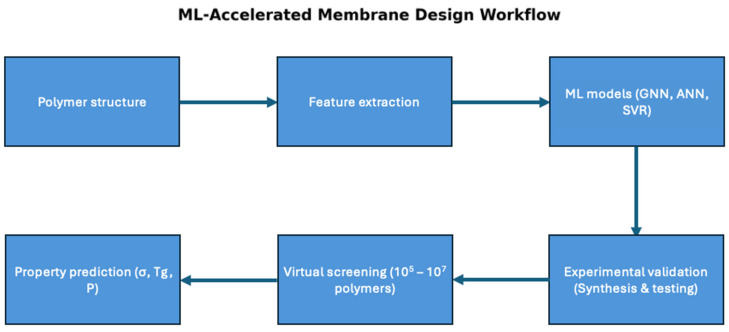
ML-based membrane workflow.

The field of ML has been applied to different fields in materials science and sustainability [74]. This impact has produced great potential in fields such as wastewater treatment where the modification of the treatment process has garnered significant improvement in the removal of contaminants [75,76]. In particular, this has a great effect on the modification of removal processes such as membrane processes, specifically in the prediction of polymer membrane properties. The development of graph neural networks (GNNs), multitask learning frameworks, and physics-informed models made it possible to screen millions of potential polymers, greatly accelerating the development of PEMs for hydrogen fuel cells. These architectures have specific boundaries where GNNs are used to predict properties by applying molecular structures in a graph architecture [77]. GNNs operate well within the operating conditions of the training data with uncertainties in extending it towards new operating conditional regimes. Multitask learning improves performance for properties that are consistent with physical correlations, but uncorrelated properties can reduce accuracy [78]. Physics-informed constraints can be bound towards realistic conditions, but there are limited assumptions and models. Figure 4 shows the workflow of this process, where the features are extracted based on the polymer structure. Then, the ML model architecture is selected for experimental validation. After virtually screening many polymers, their parameters can be predicted.

### 4.1. Predicting Polymer Properties

The prediction of polymer properties is an essential component for improving and optimizing polymer membranes. Proton conductivity remains a critical property for fuel cell membranes. The Arrhenius relationship governs temperature dependence for conductivity (*σ*), as shown in Equation (1) with activation energies (*E_a_*) being around 10–20 kJ/mol for Nafion and sulfonated polyimide membranes [79].

(1) $$\sigma =\sigma_{0}e^{-\frac{E_{a}}{RT}}$$

where *σ*_0_ is the pre-exponential factor (S cm^−1^), *R* is the universal gas constant (J mol^−1^ K^−1^), and *T* is the absolute temperature (K). Proton transport is governed by interchain interactions and segregated nanophase morphology in sulfonated polyimide (SPI) membranes. Vaz et al. [80] applied random forest regression and decision tree classifiers to this membrane, enabling a cost-effective in silico design of SPI membranes [80]. Legala et al. [81] used artificial neural networks (ANNs) to predict membrane resistance and hydration levels. After hyperparameter tuning and the addition of dropout regularization, the ANN achieved an R^2^ of 0.99. This study also showed that physics-based simulation data can be used to train data-based models to reduce laboratory experimentation. In HT-PEMFCs with phosphoric-acid-doped polybenzimidazole (PBI) membranes, a support vector regression (SVR) machine model is used based on ten input parameters [82]. These input parameters include hydrogen and oxygen stoichiometric ratios, temperature, pressure, membrane, and ionomer ion-exchange capacity (IEC) values, membrane and ionomer thicknesses, CO/H_2_ ratio, and platinum loading. The addition of optimization algorithms to the support vector regression machine makes it possible to explore the design space of HT-PEMFC without extensive experimental trials.

**Figure 5:**
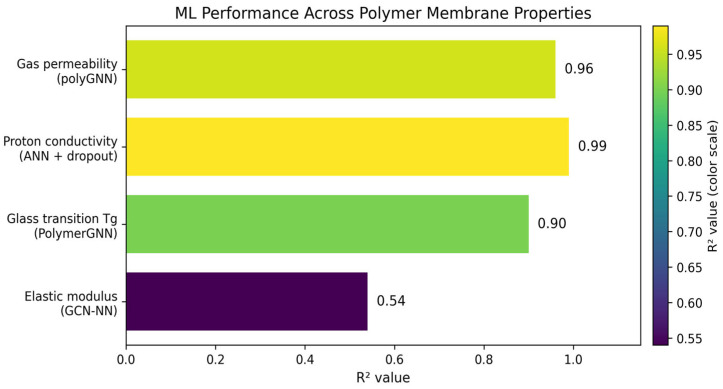
ML R^2^ comparison based on polymer membrane properties for the polyGNN framework, ANN + dropout, PolymerGNN, GCN-NN model.

Iterative synthesis–characterization cycles have been the traditional methodology to develop polymer membranes. This process requires large amounts of experimentation and the optimization of multivariable properties such as proton conductivity, gas permeability, and durability [83]. The developments in machine learning enable property prediction from molecular structures, with graph neural networks achieving coefficient of determination (R^2^) values of 0.90–0.96 across multiple properties simultaneously. Deep learning and physics-informed models can obtain correlations for membrane water content, proton conductivity, and thermal management [84]. The ML enables material selection optimization, cell design improvements, and real-time health monitoring, modifying current membrane developments [84]. A polyGNN framework has been developed by Phan et al. [85] to predict gas permeability, diffusivity, and solubility for six gases (He, H_2_, O_2_, N_2_, CO_2_, CH_4_). This approach fuses experimental data with molecular dynamics simulations, where single-task models had an R^2^ = 0.57; however, multifidelity data fusion improved this to R^2^ = 0.96. The use of physics-informed constraints, such as the use of the solution–diffusion relationship, enforces physical consistency and improves prediction reliability. GNNs have emerged as the dominant architecture for polymer property prediction because they naturally represent molecular structure as atom-node graphs with bond-edge connections [86]. The PolymerGNN has been developed, which combines graph attention network (GAT) and GraphSAGE layers with self-attention pooling to predict glass transition temperature (Tg) and inherent viscosity [87]. This model achieved R^2^ = 0.90 for Tg prediction with root mean square error (RMSE) of 30 K and mean absolute error (MAE) of 23 K, outperforming traditional fingerprint-based representations. This performance is attributed to the combination of acid and glycol monomer embeddings to enable accurate predictions for both linear and branched polymer architectures. A GCN-based model was implemented to capture features that govern thermal and mechanical properties [88]. A PoLyInfo database with 2687 polyamide structures enabled the GCN-NN model to predict the glass transition and melting temperatures. The R^2^ value was used to compare the different models and their respective properties. This metric is the coefficient of determination and explains how well the model shows the variation in the dependent variable. This metric ranges between 0 and 1, with a value of 1 showing a perfect fit and a value of 0 showing no fit. This resulted in the glass transition and melting temperature having an R^2^ of 0.90 and 0.76 and RMSE of 30 K and 40 K, respectively. The density and elastic modulus were also predicted, but with less accuracy, with an R^2^ of 0.70 and 0.54, respectively. The decrease in accuracy is attributed to the complexity of morphological factors, leading to lower accuracy. Figure 5 summarizes the results of those studies by displaying the R^2^ across polymer membrane properties revolving around gas permeability, proton conductivity, glass transition, and elastic modulus) polyGNN framework [85], ANN + dropout [81], PolymerGNN [87], GCN-NN model [88].

Molecular features govern membrane performance with quantitative structure–property relationship (QSPR) models providing possible avenues in interpreting results [89]. Yang et al. [90] screened over 9 million hypothetical polymers using ensemble deep neural networks (DNNs) with Morgan fingerprints, identifying thousands of candidates exceeding the Robeson upper bound for gas selectivity. The SHapley Additive exPlanations (SHAP) analysis revealed that methyl groups, ether linkages, and bulky pendant groups increase permeability, while oxygen-containing functional groups (carbonyl, sulfone, ether) preferentially decrease N_2_ and CH_4_ permeability relative to O_2_ and CO_2_, which enhances selectivity [90]. Phan et al. [85] pioneered path-based topological fingerprinting for polymer property prediction using Gaussian process regression (GPR). This approach identified 100 polymers more than the Robeson upper bound, as seen in Equation (2), which shows the permeability–selectivity trade-off. This identification is based on ether bonds, nitrogen-containing rings, and sulfur groups. Shastry et al. [91] used random forest and extreme gradient boosting (XGBoost) with SHAP analysis, which revealed that, despite the high predictive accuracy, these models can suffer from feature importance misattribution.

(2) $$\log\alpha \frac{A}{B}=logk-n\times logP_{A}\;$$

In Equation (2), *α* represents selectivity and *n* is a slope parameter determined by penetrant molecular sizes [92]. The integration of machine learning with molecular dynamics simulations addresses the timescale limitations of ab initio methods while maintaining quantum mechanical accuracy [93]. A study demonstrated universal neural network potentials (NNPs) for simulating proton conductivity in alkyl sulfonated polyimides at various hydration levels [94]. Planar polyamide-based acid-salt (ASPI) architectures achieved 0.2 S/cm conductivity compared to 0.03 S/cm for bent configurations, with the NNP successfully reproducing experimental conductivity differences. The simulations revealed that molecular order rather than sulfonic acid deprotonation dominates conductivity, and this finding cannot be obtained from experimental measurements, showing the benefit of these simulations. Different ML architectures are developed to combat specific simulation challenges [95]. The convolutional neural network (CNN) is based on X-ray tomography image segmentation to extract GDL porosity and tortuosity, long short-term memory (LSTM) for flooding fault detection and degradation prediction, and an ANN for membrane water content prediction. This shows the validity of ML architectures in different levels of membrane properties for transport phenomena. Membrane durability is an essential parameter that impacts fuel cell commercialization. The XGBoost was used to develop Smart Prediction of Advanced Research on PEMs (SPARK), which optimizes performance and chemical durability in ceria-containing PFSA membranes [66]. This model had a precision value that exceeded 0.9 in predicting decay rates and the optimization of ceric oxide (CeO_2_) concentration, which balances the performance and durability trade-off. Other implementations to assess conduction efficiency use the least absolute shrinkage and selection operator (LASSO), ANN, and random forest based on ten different base polymers [96]. This predicts proton conductivity (σ), hydration number (λ), and the critical conduction efficiency ratio (σ/λ), while identifying that the conduction efficiency is mostly affected by polymer crystallinity and melting point. Table 2 summarizes the aforementioned dataset size and type, ML method, the target, and performance.

**Figure 6:**
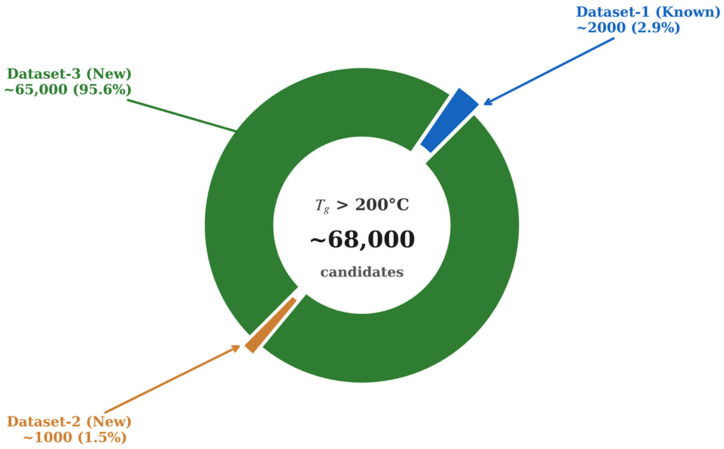
DNN fingerprint performance on the 3 datasets represented with the values representing the new candidates discovered per dataset. Redrawn using Data obtained from Tao et al. [100].

Other approaches towards the prediction of proton conductivity are based on using transformer models. Han et al. developed descriptor-based models combined with transformer transfer learning that achieved a mean absolute error of 0.91 for predicting proton conductivity [99]. This rate indicates that predictions fall within one order of magnitude of experimental values. In the prediction of thermal and chemical stability, molecular descriptors are the most important factor as they interpret the model and provide the generalization capability. Morgan fingerprints are the dominant representations, which are implemented with radius 2 and 1024–2048 bits using RDKit [100]. Tao et al. [100,101] used the glass transition temperature of polymers (T_g_) to show how machine learning can benchmark models and discover materials at high temperatures. The first study assembles three datasets from PoLyInfo and Pi1M, which have 6923 homopolymers with real T_g_, 5690 real polymers without T_g_, and 1 million hypothetical polymers [100]. The Morgan-fingerprint-based DNN maintains reasonable accuracy when it is tested on unseen real polymers and an external experimental set. Figure 6 provides a useful visualization of the fingerprint DNN’s performance on the predicted T_g_ distributions for all three datasets.

A follow-up study benchmarks 79 different models under a controlled setting on the same T_g_ task [101]. The structural unit, feature type, and learning algorithm are varied to create multiple combinations, which are evaluated on a large experimental dataset, plus a molecular-dynamics-based generated dataset. The results of the study provide a benchmark for future studies that can use it as a reference for polymer informatics using machine learning, specifically when using Morgan fingerprints. Several studies utilize descriptor-based QSPR as well to improve interpretability and to keep competitive accuracy. Casanola-Martin et al. [102] assembled 902 homopolymers with experimental T_g_ from curated literature sources and then computed 2883 Dragon descriptors, which are then reduced to 15 features using a genetic-algorithm-based multiple linear regression (GA-MLR) pipeline. The authors first fit an interpretable MLR model that explains 65% of the variance in log T_g_, then use the same 15-dimensional space to train several regressors. A support vector machine (SVM) model trained on these descriptors reaches an R^2^ value of 0.81 on the training set and 0.77 on the test set with an RMSE of 0.06 in log T_g_, and it maintains an R^2^ of 0.71 on a completely external set of 28 commercially available precursors, which demonstrates a clean trade-off between mechanistic interpretability and predictive power. In a similar approach, Afsordeh and Shirali [103] utilize a few descriptors but create an adjacency matrix that represents different polymer structures collapsed into four structural features based on flexibility, side-chain occupancy length, polarity, and hydrogen-bonding capacity, respectively. Using this foundation, they benchmark several regressors and report extra trees reaching an R^2^ of 0.97, and using feature importance analysis, they conclude that flexibility is the most dominant descriptor out of all. Yu et al. [104] use the descriptor-based approach with a much larger and more heterogeneous dataset with the random forest technique and reach an R^2^ of 0.86. The study increases T_g_ by stiffening chains and strengthening intermolecular interactions, whereas rotatable bond counts and bulky flexible side groups lower T_g_ by enhancing conformational freedom.

For chemical stability prediction, a study by Zou et al. [105] looked at combining anion exchange membranes (AEMs) with ANN methods where they were trained on Hammett substituent constants to predict OH^−^ attack resistance and achieved 99.78% accuracy. Yuan et al. [106] applied ML for screening membrane backbones from millions of hypothetical AEM candidates and demonstrated its feasibility. A study used physics-enforced neural networks (PENNs), which combine polymer genome fingerprints with degradation equations, to predict hydroxide conductivity decay over approx. 10,000 h using only limited early-time experimental data [107]. Genetic algorithms (GAs) can be ensembled with up to twelve ML models that were used to high-throughput screen over 172 million hypothetical copolymers. Here, 2519 candidates were identified from this set through a conductivity–dimensional stability trade-off metric [108]. Batra et al. [109] developed random forest and gradient boosting classifiers trained on 207 metal organic framework (MOF) structures using metal node features (e.g., atomic radius, ionization potential), organic ligand descriptors (MQNS30, MQNS36), and metal–ligand molar ratios to classify MOFs as stable or unstable in aqueous media. Nandy et al. [110] expanded this data landscape through the MOFSimplify database that includes 3132 structures with thermal stability labels and 2179 with solvent-removal stability data. Revised autocorrelation (RAC) fingerprints and geometric features from Zeo++ were used in artificial neural networks that achieved 0.76 accuracy and 0.79 ROC-AUC for solvent-removal stability. A 47 °C mean absolute error was reported for decomposition temperature prediction. These studies show the success of using ML methods to assess polymer properties in terms of conductivity, thermal, and chemical properties. However, further considerations are needed in terms of dataset, scalability, and generalizability, despite the initial success.

### 4.2. Hydration Dynamics and Flow

**Figure 7:**
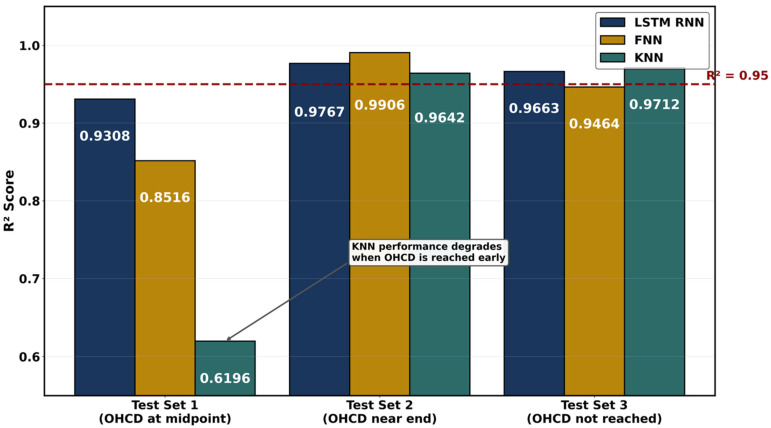
The coefficient of determination (R^2^) quantifying agreement between model-predicted and experimental high-frequency resistance (HFR) across three hold-out test sets. Test Set 1 included OHCD at the midpoint, Test Set 2 near the end, and Test Set 3 did not reach OHCD. Redrawn using data obtained from Paciocco et al. [113].

PEMFCs are met with water management challenges wherein membrane hydration directly governs proton conductivity [111], and excess water causes catastrophic flooding. The ML approaches predict these complex multiphase phenomena with accuracy exceeding 98% while reducing computational costs by orders of magnitude compared to traditional computational fluid dynamics [81]. Neural networks have the potential to conduct molecular dynamics simulations of alkyl sulfonated polyimide membranes across hydration levels λ = 3–15 that revealed planar backbone structures achieve conductivities of 0.2 S/cm compared to 0.03 S/cm for bent configurations [94]. Random forest regression was used to optimize the conductivity-to-hydration-number ratio (σ/λ) in graft-type membranes, identifying crystallinity and melting point as influential base polymer properties for high proton conduction efficiency at low water content [96]. Artificial neural networks with dropout regularization predict membrane hydration level λ [81] with R^2^ greater than 0.99 from operating parameters including current, temperature, pressure, and relative humidity. An ANN surrogate model trained on 43 CFD datasets was used to optimize block channel parameters, wherein the scoring method used mass transfer and drainage performance to rank channel designs and showed 10–15% improvements in peak power density over serpentine designs [112]. Paciocco et al. [113] developed the first LSTM-based framework for predicting optimal membrane hydration. A 3.11% mean absolute percentage error was achieved for high-frequency resistance prediction, and classification precision exceeded 98% for optimal hydration current density determination using electrochemical impedance spectroscopy inputs. Figure 7 compares feedforward neural network (FNN), LSTM, recurrent neural network (RNN), and k-nearest neighbor (KNN) predictions of high-frequency resistance against experimental measurements across three hold-out test sets.

ResNet50-based convolutional neural networks applied to neutron radiography images for water areal mass density quantification achieved 95.7% accuracy [114]. This study revealed liquid water emergence occurs at approximately 55% downstream under 50% relative humidity conditions. A comparison [115] between response surface methodology, artificial neural networks, random forest, and boosting models for predicting water saturation and oxygen transport resistance revealed that CatBoost achieved optimal performance with R^2^ = 0.983 for saturation prediction and an R^2^ of 0.9899 for resistance characterization. Ko et al. [116] formulated physics-informed neural networks encoding a membrane degradation indicator and ohmic resistance directly into the loss function. Advanced hierarchical modeling by coupling three-dimensional transport equations with neural-network-based electrochemical sub-models achieved RMSE below 0.2% at 0.5% of conventional 1D computational costs, while preserving non-linear membrane hydration effects [117]. Multitask graph neural networks that learn from short (5 ns) molecular dynamics simulations can predict ionic conductivity with a mean absolute error of 0.078 log_10_(S/cm), which enables screening of over 6000 polymer electrolyte candidates [118]. Table 3 summarizes the different ML approaches below in terms of membrane, application, and performance metrics.

### 4.3. AI-Based Optimization Techniques

Aside from the prediction of polymer properties, hydration dynamics, and flow, machine learning can identify optimal loadings and cost-effective approaches. AI-based optimization reduces experimental design cycles by 40–60% while achieving prediction accuracies exceeding R^2^ = 0.95 for critical membrane properties, including proton conductivity, water uptake, and mechanical stability [120]. The optimization of nanoparticle loading and cost performance remains integral to membrane design and optimization.

ANNs are applied to predict membrane properties as a function of nanoparticle loading to accelerate nanocomposite development. Fetanat et al. [121] demonstrated that optimized ANN models with sensitivity analysis can predict permeate flux and foulant rejection in thin-film nanocomposite membranes with high accuracy. The ANN model was able to identify the main input parameters (filler concentration and average filler size) alongside secondary parameters (input parameters alongside polymer type, polymer concentration, solvent characteristics, and contact angle). In PEMFCs, it is established that ML models trained on experimental and simulation-based datasets can reduce experimental trial and error costs while targeting the output effectively [120]. Gradient boosting tree models have been used alongside neural networks to relate the nanoparticle loading and membrane properties [58]. This model has been used to identify the primary factors that affect membrane permeability, with nanoparticle loading, pore size, and hydrophilicity being the primary factors. The study reports that the optimal nanoparticle loadings of 0.5–2 wt.% for graphene oxide in Nafion membranes and 6 wt.% for strontium titanate (SrTiO_3_) in sulfonated poly(ether ether ketone) (SPEEK)/polyamide-imide (PAI) composites achieved 10.78 × 10^−3^ S cm^−1^ at 150 °C and 10 wt.% for silica in SPEEK/poly(vinyl alcohol) (PVA) achieved 0.65 S/cm at 80 °C. To optimize performance, multiple considerations are needed in terms of fuel cell performance, durability, and manufacturing cost. This compels the use of a multiobjective optimization framework [122]. The multiobjective optimization problem for membrane design is based on the minimization of Equation (3) subject to *gⱼ*(*x*) ≤ 0, *j* = 1, 2, …, *p* and *hₖ*(*x*) = 0, *k* = 1, 2, …, *q*, where *xᵢᴸ* ≤ *xᵢ* ≤ *xᵢᵁ*.

(3) $$F\left(x\right)=\left[f_{1}\left(x\right),\;f_{2}\left(x\right),\ldots \ldots .,\;f_{m}\left(x\right)\right]$$

In Equation (3), *x* represents the decision variable vector (nanoparticle loading, operating conditions, structural parameters), *fᵢ*(*x*) represents objective functions (power density, efficiency, cost), and constraints define feasible operating and manufacturing bounds. Multiple variance analysis and the non-dominated sorting genetic algorithm (NSGA-II) are used to simultaneously optimize power density, system efficiency, and oxygen distribution uniformity [122]. The devised framework reduced the number of decision variables from 11 to 6 via variance analysis, with the algorithm being used to motivate optimization. The optimal solution had a power density of 0.6327 W·cm^−2^, system efficiency of 26.16%, and exergy efficiency of 43.94%. Compared to the baseline design, this framework improved the results by 13.18%, 7.06%, and 20.29% for the power density, system efficiency, and exergy efficiency, respectively. A modification of this framework applied an Adam-optimized backpropagation neural network with NSGA-II [123]. This modification resulted in improvements of 27% in power density, 9.7% in system efficiency, and 6.6% in oxygen distribution uniformity compared to baseline conditions across seven operating parameters. Another study applied multiobjective optimization using the Pareto optimal frontier (POF) coupled with the analytic hierarchy process (AHP) and a decision-making method to evaluate efficiency under the consideration of levelized cost of electricity, size, and greenhouse gas emissions [124]. The result showed that a higher efficiency is connected to a higher levelized cost of electricity, leading to improvements of 9.93% for efficiency, 16.95% for levelized cost, 37.13% for size, and 7.77% for emissions compared to the baseline [125]. Additional optimization methods include Bayesian optimization combined with an ANN to identify optimal gas diffusion. This architecture leads to a minimum number of iterations to achieve the optimum results. After 40 iterations, the power density is 2.17 W cm^−2^, and the limiting current density is approximately 7200 mA cm^−2^ [125]. Compared to traditional approaches, this configuration achieved a 2.7× improvement, showing the advantage of combining multiple approaches [125].

Currently, platinum catalyst loadings have the largest cost drivers in PEMFC systems, facilitating the need to improve performance. AI-based methods have been used in the optimization of low-platinum formulations to improve performance. Yin et al. employed machine learning to quantify the impact of chemical ordering on thermodynamic stability in platinum–cobalt intermetallic catalysts [126]. The addition of Cu or Ni stabilizes the energy while forming trade-offs between specific activity and active surface area. The resulting ternary Pt_2_CoCu and Pt_2_CoNi catalysts achieved peak power density of approximately 1.1 W cm^−2^ at ultralow loadings of 0.056 mgPt cm^−2^ (cathode) and 0.020 mgPt cm^−2^ (anode). By comparing this performance to Pt/C cathodes, it shows double the performance of platinum loading. Initial studies demonstrated that core–shell structures with platinum–cobalt nanoparticles on PGM-free substrates could achieve mass activities of 1.08–1.77 A mgPt^−1^ [127]. This result exceeds the U.S. Department of Energy (DOE) year 2025 target of 0.44 A mgPt^−1^ while using only approximately 3% platinum by weight compared to 20–30% in commercial catalysts. The interaction between Pt-Co nanoparticles and PGM-free sites improved both activity and durability, with a voltage drop at 0.8 A cm^−2^ of only 6 mV after 30,000 cycles versus the DOE target of less than 30 mV.

In traditional optimization, response surface methodology (RSM) uses quadratic regression to model property–composition relationships, which is fitted according to Equation (4).

(4) $$y=\beta_{0}+\Sigma \beta_{i}x_{i}+\Sigma \Sigma \beta_{ij}x_{i}x_{j}+\Sigma \beta_{ii}{x_{i}}^{2}+\varepsilon$$

**Figure 8:**
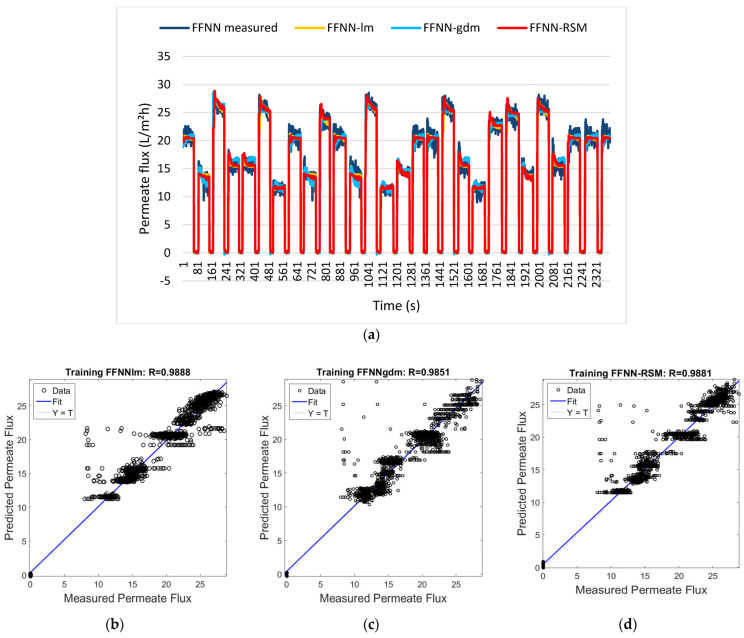
(**a**) Permeate flux for FFNN−measured, FFNN−lm, FFNN−gdm, and FFNN−RSM models. Comparison of the measured and predicted permeate flux of POME between (**b**) FFNN−lm approach, (**c**) FFNN−gdm approach, and (**d**) FFNN−RSM approach for the training dataset [129].

Applying it to the context of proton conductivity and power density optimization, y represents the response (proton conductivity, power density), *βᵢ* are linear coefficients, *βᵢⱼ* are interaction coefficients, *βᵢᵢ* are quadratic coefficients, and *ε* represents random error. Hybrid approaches have focused on combining RSM with ANNs for optimal performance [128]. Ibrahim et al. [129] demonstrated that RSM-optimized ANN via a feedforward neural network (FFNN) achieved R = 0.9866 with only 60 experimental runs in 233 s, compared to 120 runs and 543 s for conventional approaches, showing a 50% reduction in experimental resources. Compared to the standard FFNN and FFNN coupled with gradient descent and momentum (GDM), FFNN-RSM had comparable results and even outperformed FFNN-GDM while showing greater efficiency over computational resources. Figure 8a displays the training results for the permeate flux outputs for the FFNN-lm, FFNN-gdm, and FFNN-RSM models. Figure 8b–d show this performance by comparing the three configurations with the differences in the correlation coefficient being 0.98727, 0.98471, and 0.98661 for FFNN, FFNN-GDM, and FFNN-RSM, respectively. Other approaches compared AI methods such as SVR, GPR, and DNN with dropout layers [130]. The result found that DNNs achieve a mean average percentage error (MAPE) of 0.81% while SVR achieved 0.83% across seven input variables, including acid doping level, temperature, Reynolds numbers, and hydrogen mole fraction. The incorporation of physical considerations with ML can lead to optimization capabilities in deformed and graded structures [131].

## 5. Challenges and Limitations

The aforementioned studies show how effective ML models can be in learning and modeling individual properties, such as conductivity, permeability, and durability. Multitask and physics-informed models can capture the interactions between the variables, but within the limited training space. This requires a broader range of data to include greater operating regimes. There are certain challenges with the use of AI in material design. They primarily target areas such as availability, generalizability, and scalability. In ML, the presence of large datasets is essential to be able to model the full extent of the data. In the field of polymer science, there are limited experimental datasets, creating a constraint in utilizing ML to the full extent. In some cases, small datasets are used, and even though some studies present promising results, this remains a hurdle. Gao et al. [98] addressed this through self-supervised pre-training of graph neural networks on unlabeled polymer structure data. The implementation led to a reduction of RMSE by 28.39% for electron affinity and 19.09% for ionization potential predictions compared to supervised learning without pre-training. This transfer learning approach enables potentially accurate property prediction from substantially smaller labeled datasets, which are critical for specialized membrane chemistries where experimental data is scarce [132]. Therefore, to mitigate this issue, multiple datasets should be integrated to evaluate the robustness of the proposed method [98]. However, this presents a new issue regarding the differences in each dataset and how to resolve these differences during their combination. There should also be considerations regarding the complexity of polymer structures [133]. The high molecular weight and complex macromolecules of polymers cause intermolecular interactions. Unlike crystalline structures, which are simpler, these complex molecules are not easily readable to a machine, nor are they computationally efficient. The development of sophisticated computational methods rapidly increased the design space for the discovery of new polymers. However, the level of physical experimentation of these new polymers in the lab is much slower, requiring the need for an efficient and rapid method of experimentation, as this determines the validity of the training data, which is then used in ML. This shows the need to develop experimental methods, showing the need to generalize and scale those methods for effective training.

## 6. AI Integration with Green Hydrogen Economy

Artificial intelligence is becoming an important part of the future green hydrogen economy, wherein digitalization and decarbonization drivers are leading various energy policy changes worldwide. Global hydrogen demand is expected to grow fivefold by 2050, with China alone targeting 200 million metric tons annually, while Japan and South Korea collectively aim for 35 million [134]. Governments across major economies have incorporated AI-driven optimization into their national hydrogen strategies, though regional approaches differ considerably in scope and implementation pace.

**Figure 9:**
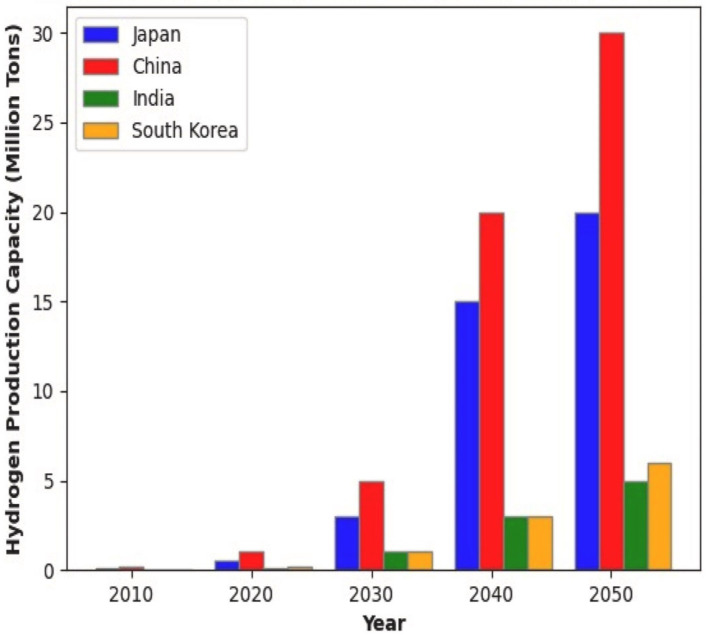
Projected green hydrogen production capacities of Asian economies in the year 2050 [135].

Raghuvanshi et al. [135] supported the correlation between AI-based optimization approaches facilitating the green hydrogen transition based on the attitudes of Asian economies, such as China, Japan, India, and South Korea, which support AI-enabled hydrogen adoption. Figure 9 shows their individual projected capacity for green hydrogen production in the year 2050, wherein the production costs are expected to decrease.

Asian economies have established differentiated pathways reflecting their energy security imperatives and industrial bases. China dominates global electrolyzer manufacturing compared to its Western counterparts, which gives it a huge boost for green hydrogen production [136]. Zhang and Wen [137] demonstrated through empirical analysis that AI enhances China’s energy security with effects more pronounced in eastern regions characterized by greater economic openness. It appears that AI and ML provided more opportunities for green hydrogen production through various processes from electrolysis to dark fermentation, for example [138,139]. The European Union on the other hand has pursued what policymakers term a “twin transition,” which focuses on the integration of green and digital transformations. Necula [140] reviewed AI applications in European clean energy technologies and found that deep learning and neural networks enhance efficiency and management of hydrogen production and other renewable energy systems. The EU’s REPowerEU initiative doubled hydrogen targets to 20 million tonnes by 2030 [141] with the European Hydrogen Bank allocating EUR 720 million in its first auction to bridge the cost gap between green and conventional hydrogen [141]. The United States’ approach centers on technology-neutral tax incentives with the Inflation Reduction Act establishing production tax credits of up to USD 3/kg for hydrogen with lifecycle emissions below 0.45 kg CO_2_e/kgH_2_, while the Department of Energy’s National Clean Hydrogen Strategy targets 10 million metric tons by 2030 [142]. Notably, the American framework is less lenient than European standards, defining clean hydrogen with a carbon footprint of 0.06 kg CO_2_/kWh which corresponds to 2 kg CO_2_e/kgH_2_ compared to the EU’s lenient 3.38 kg CO_2_e/kgH_2_ threshold [143].

## 7. Conclusions

Machine learning has matured from a proof-of-concept tool to an essential methodology for accelerating polymer membrane development. Graph neural networks now routinely achieve R^2^ > 0.90 for transport property prediction, while multitask learning frameworks exploit physical relationships to enhance accuracy further. The NSGA-II combined with neural networks or ensemble learning surrogates has emerged as the dominant framework, achieving improvements of 10–30% across multiple performance metrics while reducing experimental requirements by 40–60%. Bayesian optimization shows particular promise for expensive evaluations, identifying optimal solutions in as few as 40 iterations. The integration of ML with molecular dynamics simulations provides atomic-resolution outcomes at efficient computational costs, and self-supervised learning addresses the persistent challenge of limited experimental data. However, issues regarding data availability, scalability, and generalizability still persist and require more thorough investigation for effective use. The implications of using ML on polymers, specifically hydrogen fuel cell applications, lead to faster identification of membrane candidates with optimized proton conductivity, gas barrier properties, and durability. These potential benefits can result in a cost-effective way to commercialize fuel cells. These models show how effective they are in capturing the synergistic effect for the different properties across the design board. In terms of translating these benefits to industrial scales, these AI approaches and methods can be integrated at the membrane manufacturing level. At the research stage, property prediction methods can virtually screen candidates before synthesis. At the pilot scale, methods such as Bayesian and multiobjective frameworks can guide formulation decisions. Full-scale deployment would require standardized data collection protocols, digital twin integration with process control systems, and workforce training in ML model interpretation and maintenance.

## Figures and Tables

**Table 2:** Summary of ML methods and performance.

Dataset Size	Polymer Type	ML Method	Target Property	Best Performance	Ref.
820 polymers	General	polyGNN (multitask)	P, D, S for 6 gases	R^2^ = 0.96	[85]
296 polyesters	Polyesters	PolymerGNN (GAT + GraphSAGE)	Tg, inherent viscosity	R^2^ = 0.90	[87]
2687 polymers	Polyamides	GCN-NN	Tg, Tm, ρ, E	R^2^ = 0.90 (Tg)	[88]
CFD data	PEM	ANN with dropout	Membrane resistance	R^2^ ≥ 0.99	[81]
~500/gas	PIMs, polyimides	DNN ensemble	Gas permeabilities	R^2^ = 0.85–0.92	[90]
~11,000	General	GPR	P, D, S	>100 above bound	[83]
Literature	SPIs	Random forest	Proton conductivity	High classification	[80]
Experimental	AEMs	FCNN	Ionic conductivity	180,000 screened	[97]
Experimental	PFSA + ceria	XGBoost	Durability, voltage	Precision > 0.9	[66]
Pre-trained	General	Self-supervised GNN	Electron affinity, ionized potential	28% RMSE reduction	[98]

**Table 3:** Summary of ML approaches for hydration.

ML Method	Membrane/System	Application	Performance Metrics	Ref.
ANN with dropout	Nafion PEMFC	Membrane hydration level	R^2^ ≥ 0.99	[81]
Neural network potential	Alkyl sulfonated polyimides	Proton conductivity vs. hydration	σ = 0.2 S/cm (planar), 0.03 S/cm (bent)	[94]
Random forest	Graft-type PEMs	σ/λ optimization	Feature importance ranking	[96]
LSTM	PEM fuel cell	Optimal hydration prediction	MAPE = 3.11%, precision > 98%	[113]
ResNet50 CNN	PEMFC	Neutron radiography analysis	95.7% classification accuracy	[114]
CatBoost ensemble	Generic PEMFC GDL	Water saturation prediction	R^2^ = 0.983	[115]
LSTM + CNN ensemble	PEMFC	Flooding/drying pre-diagnosis	30 s predictive horizon	[119]
PINN	PEMFC stack	Degradation prognosis	Reduced data requirements	[116]
NN-driven 3D + 1D	Complete PEMFC	Multiscale modeling	RMSE < 0.2%, 0.5% compute cost	[117]
Multitask GNN	Polymer electrolytes	Conductivity screening	MAE = 0.078 log_10_(S/cm)	[118]

## Data Availability

All data are included and available in the article.

## Abbreviations

AEMs	Anion Exchange Membrane
AHP	Analytic Hierarchy Process
AI	Artificial Intelligence
ANNs	Artificial Neural Networks
BPs	Bipolar Plates
CeO_2_	Cerium Dioxide (Ceric Oxide)
CFD	Computational Fluid Dynamics
CL	Catalyst Layer
CNN	Convolutional Neural Network
DNNs	Deep Neural Networks
DOE	Department of Energy
ePTFE	Expanded Polytetrafluoroethylene
EU	European Union
Fe_3_O_4_	Iron Oxide (Magnetite)
FFNN	Feedforward Neural Network
FNNs	Feedforward Neural Networks
GAs	Genetic Algorithms
GAT	Graph Attention Network
GCN	Graph Convolutional Network
GDLs	Gas Diffusion Layers
GDM	Gradient Descent and Momentum
GNNs	Graph Neural Networks
GO	Graphene Oxide
GPR	Gaussian Process Regression
HFR	High-Frequency Resistance
HT-M	High-Temperature Membrane
HT-PEMFC	High-Temperature Proton Exchange Membrane Fuel Cell
IEC	Ion Exchange Capacity
KNN	K-Nearest Neighbor
LASSO	Least Absolute Shrinkage and Selection Operator
LSTM	Long Short-Term Memory
MAE	Mean Absolute Error
MAPE	Mean Absolute Percentage Error
MEA	Membrane Electrode Assembly
ML	Machine Learning
MLR	Multiple Linear Regression
MOF	Metal Organic Framework
NNP	Neural Network Potential
NNs	Neural Networks
NSGA-II	Non-Dominated Sorting Genetic Algorithm II
OCV	Open-Circuit Voltage
OH	Hydroxyl
OHCD	Optimal Hydration Current Density
OOH	Hydroperoxyl
PAI	Polyamide-imide
PBI	Polybenzimidazole
PEM	Proton Exchange Membrane or Polymer Electrolyte Membrane
PEMFCs	Proton Exchange Membrane Fuel Cells
PENN	Physics-Enforced Neural Network
PFSA	Perfluorosulfonic Acid
PGM	Platinum Group Metal
PIMs	Polymers of Intrinsic Microporosity
POF	Pareto-Optimal Frontier
Pt	Platinum
PVA	Poly(vinyl alcohol)
QSPR	Quantitative Structure–Property Relationship
R^2^	Coefficient of Determination
RAC	Revised Autocorrelation
RMSE	Root Mean Square Error
RNN	Recurrent Neural Network
ROS	Reactive Oxygen Species
RSM	Response Surface Methodology
SGO	Sulfonated Graphene Oxide
SHAP	SHapley Additive exPlanations
SiO_2_	Silicon Dioxide
SPARK	Smart Prediction of Advanced Research on PEMs
SPEEK	Sulfonated Polyether Ether Ketone
SPI	Sulfonated Polyimide
SrTiO_3_	Strontium Titanate
SVM	Support Vector Machine
SVR	Support Vector Regression
Tg	Glass Transition Temperature
WO_3_	Tungsten Trioxide
XGBoost	Extreme Gradient Boosting
ZrO_2_	Zirconium Dioxide
